# Cariprazine efficacy in bipolar I depression with and without concurrent manic symptoms: post hoc analysis of 3 randomized, placebo-controlled studies

**DOI:** 10.1017/S1092852919001287

**Published:** 2020-08

**Authors:** Roger S. McIntyre, Trisha Suppes, Willie Earley, Mehul Patel, Stephen M. Stahl

**Affiliations:** 1 Mood Disorders Psychopharmacology Unit, University Health Network, Toronto, Ontario, Canada; 2 Department of Psychiatry & Behavioral Sciences, Stanford University School of Medicine, Stanford, California, USA; 3 Bipolar and Depression Research Program, VA Palo Alto Health Care System, Palo Alto, California, USA; 4 Clinical Development, Allergan, Madison, New Jersey, USA; 5 Medical Affairs, Allergan, Madison, New Jersey, USA; 6 Department of Psychiatry, University of California, San Diego, California, USA

**Keywords:** Atypical antipsychotics, bipolar depression, cariprazine, dopamine D_3_ receptor, mixed features, mixed depression, mixed symptoms.

## Abstract

**Objective.:**

Mixed presentations, defined by simultaneous occurrence of depressive and manic symptoms, are difficult to treat. Antidepressants, although commonly used, have weak evidence of efficacy and may increase risk of mood destabilization. The aim of this pooled post hoc analysis was to evaluate the efficacy of cariprazine in the treatment of bipolar depression with or without concurrent manic symptoms.

**Methods.:**

Patients from 3 randomized, double-blind, placebo-controlled studies who met DSM-IV-TR or DSM-5 criteria for bipolar I disorder with a current major depressive episode were identified to have concurrent manic symptoms by baseline Young Mania Rating Scale total score ≥4. Efficacy was assessed in cariprazine 1.5 and 3 mg/day dose groups versus placebo; analyses included the least squares mean change from baseline to week 6 in Montgomery-Åsberg Depression Rating Scale (MADRS) total score.

**Results.:**

Of 1383 patients randomized to treatment, 808 (58.4%) had concurrent manic symptoms. For patients with manic symptoms, mean reduction in MADRS total score from baseline to week 6 was significantly greater for both cariprazine 1.5 and 3 mg/day compared with placebo, with least squares mean differences (LSMDs) versus placebo of −2.5 (*p* = .0033) and −2.9 (*p* = .0010), respectively; for patients without manic symptoms, the LSMD was significant for 1.5 mg/day (−3.3; *p* = .0008), but not for 3 mg/day (−1.9; *p* = .0562).

**Conclusion.:**

The results of this post hoc analysis suggest that cariprazine may be an appropriate treatment option for patients with bipolar I depression with or without manic symptoms, with higher doses potentially more effective in patients with manic symptoms.

## Introduction

Bipolar disorder is a complex and chronic mood disorder characterized by alternating or intertwining episodes of mania, hypomania, and depression.^1^ Of these mood states, the depressive phase is the most enduring and disabling feature of the disorder as patients with bipolar disorder spend up to half of their illness lifetime with depressive symptoms, approximately three times more than their time with manic/hypomanic symptoms.^2^ ^,^^3^ Further, bipolar and unipolar depression is strongly and consistently associated with increased work loss and decreased productivity^4^; this association is less defined with mania/hypomania.^5^ Treatment of bipolar depression remains a challenge for clinicians, especially given that there are fewer treatment options available for treating bipolar depression compared with mania.^2^ In addition, patients with bipolar depression can also suffer from mixed symptoms^1^ that can further complicate treatment.

The criteria for mixed episodes have changed over time. The *Diagnostic and Statistical Manual of Mental Disorders*, 4th edition (DSM-IV-TR)^6^ required the contemporaneous presence of a threshold manic and major depressive episode; however, this definition was found to be too restrictive as it did not reflect the range of presentations for bipolar disorder and excluded patients who were experiencing either a major depressive, manic, or hypomanic episode with varying degrees of subsyndromal symptoms of the opposite mood state.^7-9^ To be more clinically relevant, the definition was broadened in the *Diagnostic and Statistical Manual of Mental Disorders*, 5th edition (DSM-5)^10^ with the “with mixed features” specifier, defined as patients meeting criteria for either a major depressive, manic, or hypomanic episode with 3 or more symptoms (without meeting the full criteria as previously required by DSM-IV-TR) of the opposite mood state. Using various modified DSM-5 definitions, it has been estimated that up to 40% (depending on source) of patients with bipolar disorder will experience mixed mood symptoms during manic, hypomanic, or depressive episodes.^8^ ^,^^9^ ^,^^11^ ^,^^12^ The presentation of bipolar depression with mixed features is complex, and there is debate in the literature on whether the symptoms of mixed features are fully captured by the DSM–5 diagnostic criteria.^13-15^ It should be noted that a number of studies have suggested that the presence of as few as 3 symptoms or less may be sufficient to define a mixed population^12^ ^,^^16^; as such, the criteria for mixed features remains an iterative process.

Bipolar depression with mixed symptoms is associated with greater symptom severity, higher rates of mood episode recurrence and comorbidities, worse clinical outcomes, lower rates of treatment response, and increased risk of suicidality.^17^ Currently, no agent has been approved by the FDA or EMA for the treatment of patients with bipolar disorder exhibiting a mixed features specifier. In the absence of approved treatments for mixed features, antidepressants are commonly used to treat mixed symptoms, but they are generally ineffective and may raise the risk of manic episodes in this population.^18^ ^,^^19^ In addition, as bipolar depression is frequently misdiagnosed as unipolar depression^20^, antidepressants may also be incorrectly prescribed, which can lead to reduced treatment response. There is evidence that atypical antipsychotics, which can lower the severity of both depressive and manic symptoms, may be able to treat bipolar depression with mixed symptoms.^8^ ^,^^9^ It has also been hypothesized that atypical antipsychotics that have affinity for receptors that are thought to modulate depression, such as dopamine D_3_ and serotonin 5-HT_1A_, may be effective treatments for bipolar depression.^21^ ^,^^22^ To this end, a panel of experts have recently recommended that atypical antipsychotics should be considered as the initial treatment for patients with mixed depression.^23^

Cariprazine, a dopamine D_3_-preferring D_3_/D_2_ receptor and 5-HT_1A_ receptor partial agonist, is approved for the treatment of adults with schizophrenia (1.5–6 mg/day; United States and Europe) and manic/mixed episodes associated with bipolar I disorder (3–6 mg/day; United States). Cariprazine has also been recently approved as a monotherapy for bipolar I depression (1.5–3 mg/day; United States) based on efficacy and safety/tolerability results from 3 positive phase II/III randomized, double-blind, placebo-controlled trials in patients with bipolar I depression.^24–26^ Cariprazine has demonstrated efficacy versus placebo at both poles of the bipolar spectrum and is only the second agent approved to treat episodes of both mania and depression in patients with bipolar I disorder.

The objective of this post hoc analysis was to evaluate the efficacy of cariprazine in patients with bipolar depression, with or without concurrent manic symptoms using pooled data from the 3 aforementioned clinical studies. As these 3 studies excluded patients experiencing moderate-to-severe manic symptoms, we used slightly broader criteria in our post hoc analyses to identify patients with concurrent manic and depressive symptoms (Young Mania Rating Scale [YMRS] total score ≥4) than what is specified by the DSM-5 to define mixed features.

## Methods

Post hoc analyses were performed using pooled data from 3 phase II/III randomized, double-blind, placebo-controlled trials in patients with bipolar depression (MD-56 [NCT01396447], MD-53 [NCT02670538], MD-54 [NCT02670551]). All 3 studies were conducted in compliance with the *International Conference on Harmonisation Guidances on General Considerations for Clinical Trials and Good Clinical Practice *and the* Declaration of Helsinki.* These studies were also approved by institutional review boards or ethics committees and government agencies. All participants provided written informed consent after receiving a complete description of the studies. The studies were conducted between March 2016 and January 2018 (MD-53), March 2016 and July 2017 (MD-54), and July 2011 and January 2014 (MD-56).

### Patients

These multiregional studies, conducted in the United States and 12 other countries, enrolled adult patients 18 to 65 years of age. Patients were eligible to enroll if they had a DSM-IV-TR (MD-56) or DSM-5 (MD-53 and MD-54) diagnosis of bipolar I disorder with a current major depressive episode (duration ≥4 weeks and ≤12 weeks) and met the following inclusion criteria at baseline: 17-item Hamilton Depression Rating Scale (HAMD_17_)^27^ ^–^^29^ total score ≥20; item 1 depressed mood score ≥2; Clinical Global Impressions-Severity (CGI-S)^30^ score ≥4; and YMRS^31^ total score ≤10 (MD-56) or ≤12 (MD-53 and MD-54). For studies MD-53 and MD-54, patients were also required to be currently treated as an outpatient at the time of enrollment. Other exclusion criteria included if the patient were at risk for suicide (recent suicide attempt or as judged by the investigator based on psychiatric interview or the Columbia–Suicide Severity Rating Scale [C-SSRS]^32^), or if the patient had a history of substance dependence within 6 months prior to the study.

### Study design

All 3 studies required patients to undergo screening and washout for up to 14 days; eligible patients were randomly assigned to receive 6 (MD-53 and MD-54) or 8 weeks (MD-56) of double-blind treatment with placebo or fixed doses of cariprazine (0.75 [in MD-56 only], 1.5, or 3 mg/day). In study MD-56, all patients randomized to the cariprazine treatment groups were initiated on a dose of 0.5 mg/day and the dosage was increased to 0.75 mg/day on day 3; in the 1.5- and 3-mg/day dose groups, the dosage was increased to 1 mg/day on day 5 and then to 1.5 mg/day on day 8, and in the 3-mg/day dose group, the dosage was further increased to 3 mg/day on day 15. In studies MD-53 and MD-54, all patients randomized to receive cariprazine were initiated on a dose of 1.5 mg/day; in the 3-mg/day dose group, the dosage was increased to 3 mg/day on day 15. The primary efficacy parameter in all 3 studies was change from baseline in the Montgomery-Åsberg Depression Rating Scale (MADRS)^33^ total score at week 6. The secondary efficacy parameter in all 3 studies was change from baseline in the CGI-S score at week 6. YMRS total score was also assessed and was included to determine if there was any worsening of manic symptoms during the studies.

### Definition of concurrent manic symptoms

Post hoc analyses were based on the subgroups of patients *with* concurrent manic symptoms or *without* concurrent manic symptoms. Patients were determined to have concurrent manic symptoms if their baseline YMRS total score was ≥4; patients were categorized as not having concurrent manic symptoms if they had a baseline YMRS total score <4. Previous studies have suggested that a baseline YMRS total score ≥4 defines a patient population (patients with concurrent manic symptoms) with distinct response characteristics versus patients with baseline YMRS total score <4.^34–36^

### Post hoc analyses

Efficacy versus placebo was assessed for the pooled cariprazine 1.5 and 3 mg/day fixed-dose groups. Analyses included least squares (LS) mean change from baseline to week 6 in MADRS total and individual item score, HAMD_17_ total score, CGI-S score, and YMRS total score; these efficacy parameters were all analyzed using a mixed-effects model for repeated measures (MMRM). Rates of MADRS response (≥50% improvement) and remission (total score ≤10), HAMD_17_ (total score ≤7) remission, and CGI-S remission (total score ≤2) were analyzed using logistic regression with last observation carried forward (LOCF) to impute missing values. Statistical significance was set at a level of .05. Treatment-emergent mania was defined as having YMRS total score ≥16 at any postbaseline visit.

## RESULTS

### Baseline characteristics and patient disposition

**Figure 1.**
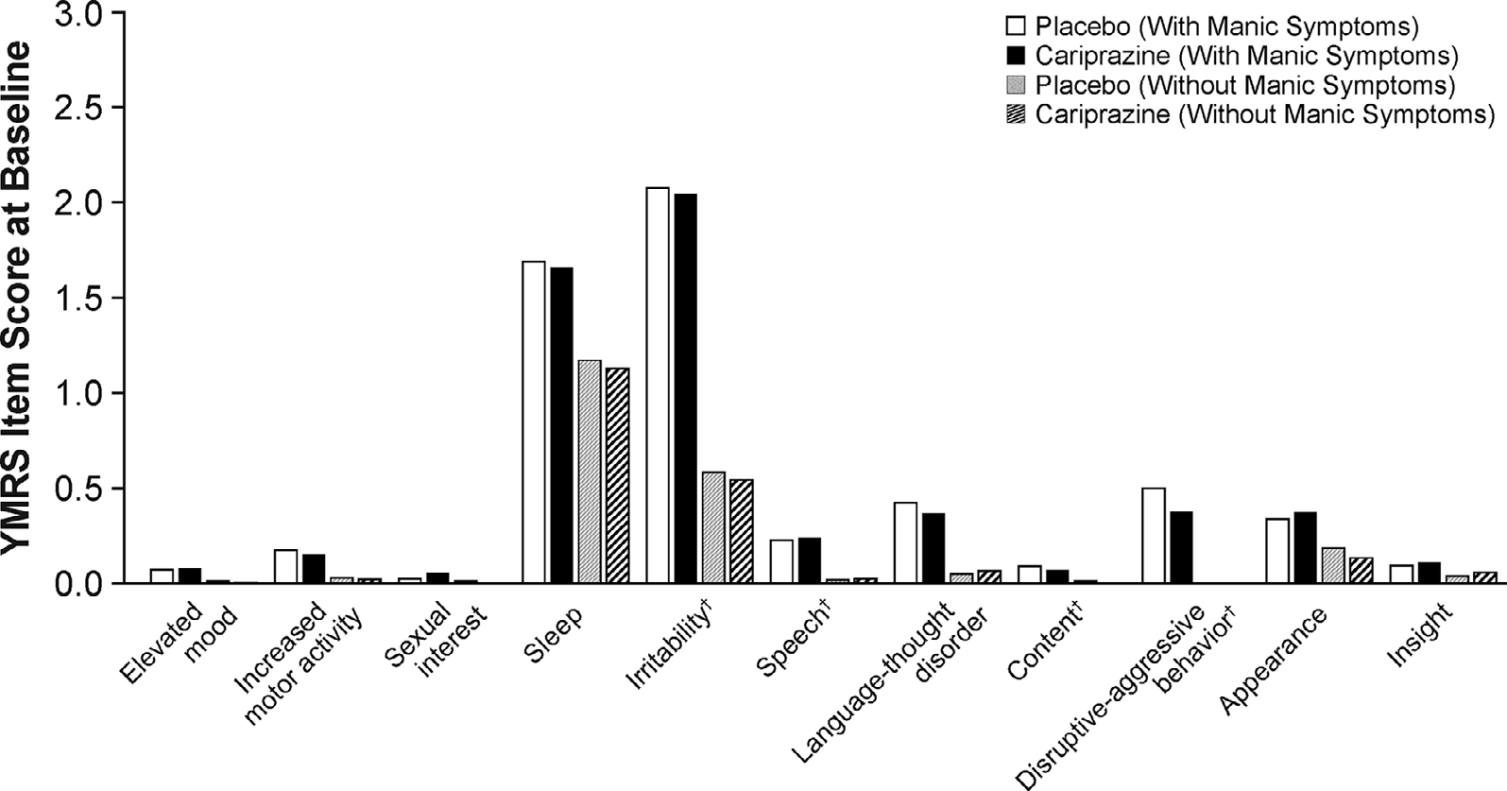
Baseline YMRS individual item scores. †Core items scored with range of 0–8; all other items scored with a range of 0–4.

A total of 1383 patients with bipolar depression were included in the ITT population; 808 patients (58.4%) were identified to have concurrent manic symptoms, and 575 patients (41.6%) did not have concurrent manic symptoms. Baseline characteristics were generally similar between patient and treatment groups except for YMRS total score, which was higher in patients with concurrent manic symptoms versus patients without concurrent manic symptoms (Table 1). The duration of current depressive episode, number of depressive and manic/mixed episodes during lifetime, and number of mood episodes (including manic, mixed, hypomanic, depressive) during the past year were also similar between patient and treatment groups (Supplemental Table 1). At baseline, individual YMRS items with the highest mean scores were the sleep and irritability items; mean baseline scores for other YMRS items were low (Figure 1).

**Table 1.** Baseline characteristics.

Characteristic	With manic symptoms (*n*=808)			Without manic symptoms (*n*=575)		
		Cariprazine			Cariprazine	
	PBO (*n* = 262)	1.5 mg/d (*n* = 275)	3 mg/d (*n* = 271)	PBO (*n* = 198)	1.5 mg/d (*n* = 186)	3 mg/d (*n* = 191)
Male, *n* (%)	102 (38.9)	102 (37.1)	108 (39.9)	86 (43.4)	65 (35.0)	75 (39.3)
Age, mean (SD), y	43.3 (11.6)	41.4 (12.1)	42.4 (11.4)	45.3 (12.5)	42.8 (11.7)	43.4 (11.5)
Race, *n* (%)						
White	190 (72.5)	202 (73.5)	201 (74.2)	152 (76.8)	144 (77.4)	150 (78.5)
Black or African–American	65 (24.8)	61 (22.2)	65 (24.0)	43 (21.7)	36 (19.4)	35 (18.3)
Other	7 (2.7)	12 (4.4)	5 (1.8)	3 (1.5)	6 (3.2)	6 (3.1)
Weight, mean (SD), kg	85.3 (21.1)	85.6 (22.2)	85.9 (19.5)	82.6 (17.9)	83.1 (19.6)	81.1 (19.5)
BMI, mean (SD)	29.8 (7.3)	29.8 (7.4)	29.9 (7.0)	28.7 (5.9)	29.0 (6.6)	28.3 (6.4)
Efficacy, mean (SD)						
MADRS total score	31.1 (4.6)	31.1 (4.5)	31.2 (4.6)	30.2 (4.4)	30.5 (4.1)	30.9 (5.0)
CGI-S score	4.5 (0.5)	4.5 (0.6)	4.5 (0.5)	4.4 (0.5)	4.5 (0.6)	4.4 (0.6)
HAMD_17_ total score	24.5 (2.8)	24.6 (3.3)	24.3 (3.1)	24.3 (2.6)	24.5 (3.1)	24.5 (3.2)
YMRS total score	5.7 (1.9)	5.5 (1.8)	5.5 (1.8)	2.1 (1.0)	2.0 (1.0)	2.0 (1.0)

Abbreviations: BMI, body mass index; CGI-S, Clinical Global Impressions-Severity Rating Scale; HAMD_17_, 17-item Hamilton Depression Rating Scale; MADRS, Montgomery-Åsberg Depression Rating Scale; PBO, placebo; SD, standard deviation; SE, standard error; YMRS, Young Mania Rating Scale.

### Efficacy on depressive symptoms

**Figure 2.**
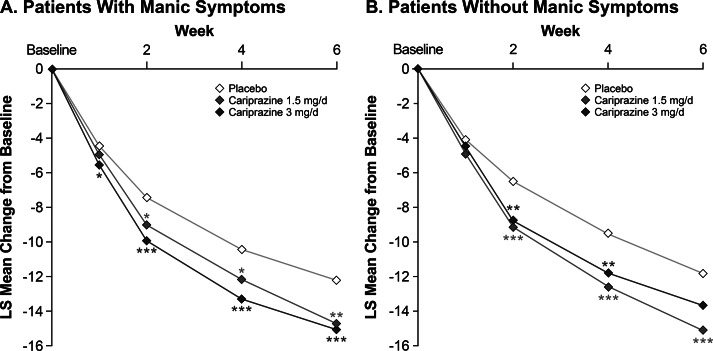
By-week change in MADRS total score from baseline to week 6 in patients (A) with or (B) without manic symptoms (MMRM). *p < .05, **p < .01, ***p ≤ .001 vs placebo. LS, least squares; MADRS, Montgomery-Åsberg Depression Rating Scale; MMRM, mixed-effects model for repeated measures.

The cariprazine treatment groups showed significantly greater improvement than the placebo group in MADRS total score beginning within the first 2 weeks and persisting to week 6 in patients with (Figure 2) and patients without (Figure 2) concurrent manic symptoms. In patients with concurrent manic symptoms, significant improvement over placebo was seen in the cariprazine 3 mg/day dose group starting at week 1 (*p* < .05). At week 6, the LS mean difference (LSMD) versus placebo was statistically significant in favor of cariprazine for all cariprazine dose groups in patients with concurrent manic symptoms and for the cariprazine 1.5 mg/day dose group in patients without concurrent manic symptoms (Table 2). On the MADRS total score change at at week 6, differences between cariprazine doses were not statistically significant in patients with (LSMD = 0.3476; *p* = .6830) and without concurrent manic symptoms (LSMD = −0.1449; *p* = .1413).

**Table 2.** Summary of Outcomes.

Outcome	**With Manic Symptoms (*n* = 808)**			**Without Manic Symptoms (*n* = 575)**		
		Cariprazine			Cariprazine	
	PBO (*n* = 262)	1.5 mg/d (*n* = 275)	3 mg/d (*n* = 271)	PBO (*n* = 198)	1.5 mg/d (*n* = 186)	3 mg/d (*n* = 191)
LSMD vs PBO at week 6, ^a^ mean (SE)						
MADRS total score	—	−2.51 (0.85)**	−2.86 (0.86)***	—	−3.30 (0.98)***	−1.85 (0.97)
CGI-S score	—	−0.24 (0.10)*	−0.25 (0.10)*	—	−0.40 (0.12)***	−0.26 (0.12)*
HAMD_17_ total score	—	−1.86 (0.65)**	−1.55 (0.67)*	—	−2.21 (0.77)**	−1.32 (0.77)
YMRS total score	—	−0.20 (0.28)	−0.37 (0.28)	—	−0.22 (0.26)	0.02 (0.26)
Response/remission at week 6, ^b^ n (%)						
MADRS responders	99 (37.8)	128 (46.6)*	135 (49.8)**	66 (33.3)	84 (45.2)*	80 (41.9)
Odds ratio	—	1.43	1.64	—	1.67	1.48
MADRS remitters	55 (21.0)	86 (31.3)**	85 (31.4)**	41 (20.7)	60 (32.3)**	48 (25.1)
Odds ratio	—	1.74	1.76	—	1.89	1.35
CGI-S remitters	60 (22.9)	83 (30.2)*	79 (29.2)*	38 (19.2)	55 (29.6)***	48 (25.1)**
Odds ratio	—	1.45	1.40	—	1.90	1.62
HAMD_17_ remitters^c^	43 (17.1)	82 (31.8)***	58 (22.3)	35 (19.3)	51 (30.0)*	40 (22.4)
Odds Ratio	—	2.26	1.42	—	1.81	1.22
Treatment-emergent mania at any postbaseline visit, *n* (%)						
YMRS total score ≥16	6 (2.3)	5 (1.8)	2 (0.7)	3 (1.5)	2 (1.1)	2 (1.0)

a Analyzed using MMRM.

b Logistic regression using LOCF.

c Number of patients included in the HAMD_17_ remission analysis were different than the ITT population (patients with manic symptoms: PBO=251, 1.5 mg/day = 258, 3 mg/day = 254; patients without manic symptoms: PBO=181, 1.5 mg/day = 170, 3 mg/day = 179).

*Note*: *p < .05, **p < .01, ***p ≤ .001 vs placebo.

Abbreviations: CGI-S, Clinical Global Impressions-Severity Rating Scale; HAMD_17_, 17-item Hamilton Depression Rating Scale; ITT, intent-to-treat; LOCF, last observation carried forward; LSMD, least squares mean difference; MADRS, Montgomery-Åsberg Depression Rating Scale; MMRM, mixed model for repeated measures; PBO, placebo; SE, standard error; YMRS, Young Mania Rating Scale.

**Figure 3.**
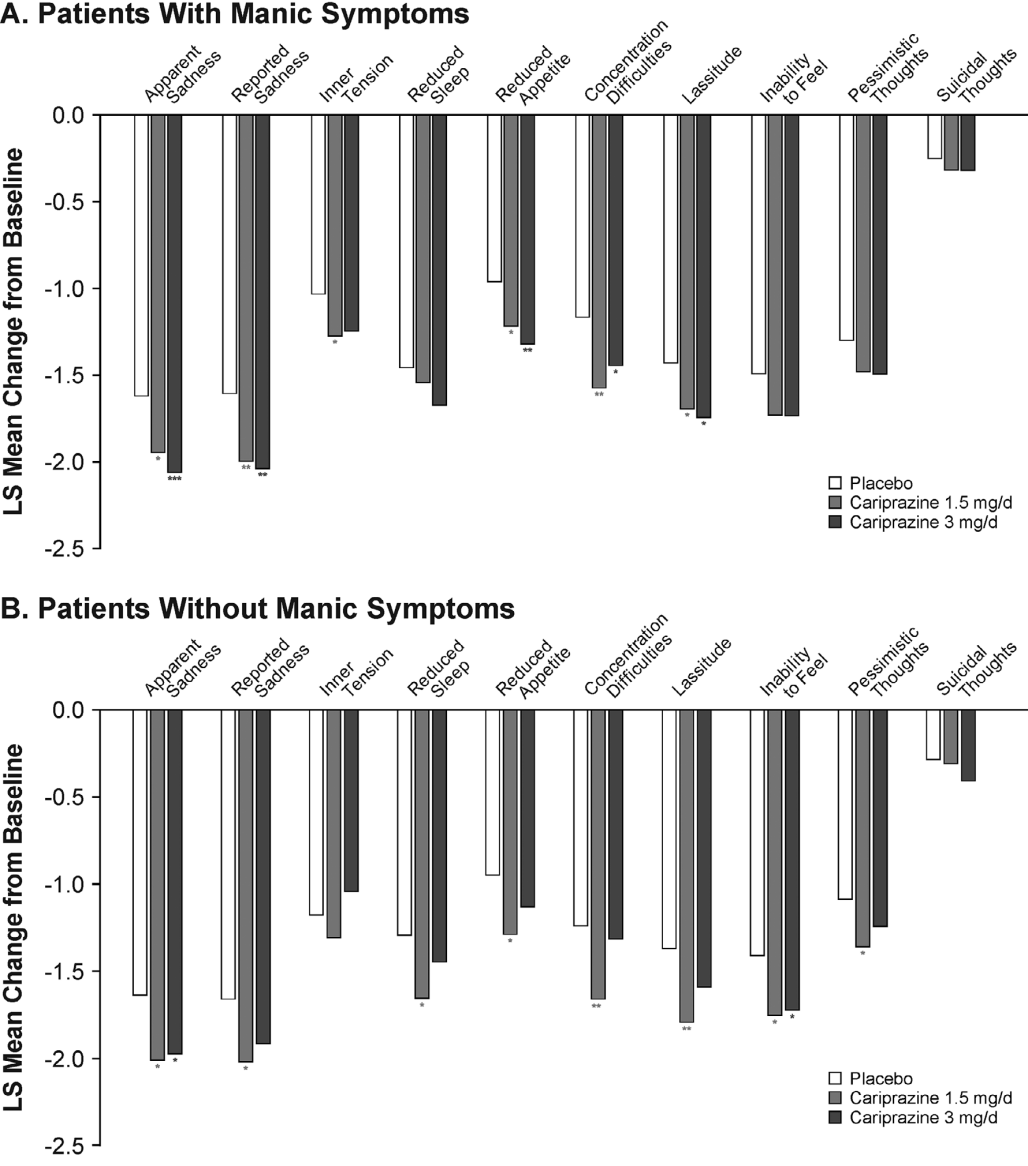
Change in MADRS individual item score from baseline to week 6 in patients (a) with or (b) without manic symptoms (MMRM). *p < .05, **p < .01, ***p < .001 vs placebo. LS, least squares; MADRS, Montgomery-Åsberg Depression Rating Scale; MMRM, mixed-effects model for repeated measures.

Cariprazine demonstrated significantly greater improvement than placebo across a range of MADRS individual items in patients with (Figure 3) and without (Figure 3) concurrent manic symptoms. There were slight differences in the number of significant items in each subgroup based on the dose of cariprazine. In patients with manic symptoms, both doses of cariprazine demonstrated efficacy versus placebo on the items of apparent sadness, reported sadness, reduced appetite, concentration difficulties, and lassitude; cariprazine 1.5 mg/day also demonstrated efficacy versus placebo on the inner tension item. In patients without concurrent manic symptoms, cariprazine 1.5 mg/day demonstrated efficacy versus placebo on 8 items (apparent sadness, reported sadness, reduced sleep, reduced appetite, concentration, lassitude, inability to feel, and pessimistic thoughts); cariprazine 3 mg/day demonstrated efficacy versus placebo on the apparent sadness and inability to feel items.

In patients with concurrent manic symptoms, the percentage of patients who met MADRS response and remission criteria at week 6 was statistically higher in all cariprazine dose groups (response: 1.5 mg/day, number needed to treat [NNT] = 12; 3 mg/day, NNT = 9; remission: 1.5 mg/day, NNT = 10; 3 mg/day, NNT = 10; all, *p* < .05) versus placebo (Table 2). In patients without concurrent manic symptoms, the percentage of patients who met MADRS response and remission criteria at week 6 was statistically higher in the cariprazine 1.5 mg/day group (response: NNT = 9; remission: NNT = 9; both, *p* < .05) versus placebo (Table 2). The rate of MADRS response and remission at week 6 was numerically greater than placebo in the cariprazine 3 mg/day group, but the differences were not statistically significant (response: *p* = .0655; remission: *p* = .2207).

The cariprazine treatment groups showed significantly greater improvement than the placebo group in HAMD_17_ total score beginning after 2 weeks of treatment and persisting to week 6 in patients with and without concurrent manic symptoms (Supplemental Figure 1, Table 2). The percentage of patients who met HAMD_17_ remission criteria at week 6 was significantly higher in favor of cariprazine versus placebo for patients with concurrent manic symptoms in the cariprazine 1.5 mg/day dose group (NNT = 7; *P* < .01) and for patients without concurrent manic symptoms in the 1.5 mg/day dose group (NNT = 10; *p* < .05). The HAMD_17_ remission rate in patients treated with cariprazine 3 mg/day were numerically greater than placebo, but the differences were not significant (Table 2). On the HAMD_17_ total score change at week 6, differences between cariprazine doses were not statistically significant in patients with (LSMD = ‑0.3108; *p* = .6350) and without concurrent manic symptoms (LSMD = ‑0.8850; *p* = .2537).

### Other efficacy parameters

All cariprazine treatment groups showed significantly greater improvement than the placebo group in the CGI-S score beginning within the first 2 weeks and persisting to week 6 in patients with and without concurrent manic symptoms (Supplemental Figure 2, Table 2). In patients with concurrent manic symptoms, significant improvement over placebo was seen in the 3 mg/day dose group starting at week 1 (*p* < .05). The percentage of patients who met CGI-S remission criteria at week 6 was statistically significantly higher in all cariprazine dose groups versus placebo (Table 2). On the CGI-S score change at week 6, differences between cariprazine doses were not statistically significant in patients with (LSMD = 0.0027; *p* = .9789) and without concurrent manic symptoms (LSMD = ‑0.1445; *p* = .2312) on the CGI-S score change at week 6.

Rates of treatment-emergent mania were low (<3%) in all treatment groups and numerically lower in the cariprazine groups compared with the placebo group (Table 2). YMRS total scores decreased in all treatment groups for patients with concurrent manic symptoms (placebo = −1.36; 1.5 mg/day = −1.56; 3 mg/day = −1.73) and for patients without concurrent manic symptoms (placebo = ‒0.13; 1.5 mg/day = −0.35; 3 mg/day = −0.11); differences between groups were not statistically significant (Table 2). On the YMRS total score change at week 6, differences between cariprazine doses were also not statistically significant in patients with (LSMD = 0.1690; *p* = .5395) and without concurrent manic symptoms (LSMD = −0.2372; *p* = .3601).

## Discussion

Mixed symptoms in bipolar depression are associated with greater symptom severity, higher rates of mood episode recurrence and comorbidities, worse clinical outcomes, lower rates of treatment response, and increased risk of suicidality.^17^ Currently, the treatment of bipolar depression with concurrent manic symptoms remains a challenge, and while antidepressants are commonly used, they are generally ineffective and may increase the risk of mood destabilization and manic episodes^18^ ^,^^19^ ^,^^34^; thus, having new effective treatment options for this patient population is imperative. On the basis of available evidence and in the absence of approved agents, a panel of experts has recently recommended that atypical antipsychotics should be considered as the initial treatment for patients with mixed depression, ^23^ though some controversary exists regarding the diagnostic criteria.^13^ ^–^^15^ Several reports have identified that the presence of 1–3 definite manic symptoms is clinically relevant.^12^ ^,^^16^ Using this broader definition of “with mixed features” in patients with bipolar depression, several studies have suggested that atypical antipsychotics may be an effective treatment in this patient population^35^ ^–^^37^; however, it should be noted that the majority of these studies have been performed post hoc.^8^ ^,^^9^ Our analysis was designed in a similar manner; to be identified as having concurrent manic symptoms, patients had baseline YMRS total score ≥4 but without requiring any minimum number of manic symptoms.

In this post hoc analysis of 3 pooled phase II/III randomized, double-blind, placebo-controlled trials in patients with bipolar depression, more than half of the patients (58.4%) met the criterion for concurrent manic symptoms (YMRS total score ≥4 at baseline); this is relatively consistent with previously reported prevalence rates for mixed symptoms, which can range from 11% to 70% depending on the study and criteria used.^8^ ^,^^9^ ^,^^11^ ^,^^12^ ^,^^16^ In patients with concurrent manic symptoms, sleep and irritability items had the highest mean baseline scores; this is consistent with previous reports as sleep and irritability have been identified to be common manic symptoms occurring in patients with bipolar depression.^12^ ^,^^16^ ^,^^38^ Previous reports have also identified that patients with bipolar depression and mixed symptoms are more likely to be female compared with patients without manic symptoms^12^ ^,^^16^ ^,^^36^; however, in our analyses, the sex distributions were similar between patient subgroups.

In patients with bipolar I depression, cariprazine demonstrated efficacy versus placebo in improving depressive symptoms in patient subgroups with and without concurrent manic symptoms as measured by MADRS and HAMD_17_ total scores. When both patient subgroups are considered, treatment effects were seen on a wide range of depressive symptoms as cariprazine was more effective than placebo on 9 of 10 MADRS individual items. The CGI-S scale, although it is not a direct measure of depressive symptoms, provides a clinician-rated view of a patient’s global functioning and takes into consideration factors such as patient history, symptoms, behavior, and psychosocial condition; overall, the CGI scale is meant to provide a useful outcome to help clinicians determine the clinical relevance of patient change. In these post hoc analyses, cariprazine significantly improved the CGI-S score versus placebo in patients with and without manic symptoms, indicating that cariprazine provided meaningful clinical benefits in patients with bipolar I depression.

On depressive symptoms, both doses of cariprazine (1.5 and 3 mg/day) were consistently more effective than placebo in patients with bipolar depression and concurrent manic symptoms, while only the 1.5 mg dose was consistently more effective than placebo in patients without manic symptoms. The reason for these differential dose effects is unknown; however, one potential explanation could be related to the pharmacology of cariprazine. In mixed presentations, affected individuals exhibit multidimensional psychopathology including cognitive dysfunction, anhedonia, and significant difficulties in affect regulation (eg, anxiety, irritability); drugs with different receptor profiles across their dosing range may have implications for treating patients with such complex symptom profiles.^39^ Pharmacodynamic studies have suggested that the receptor binding profiles for cariprazine do indeed differ depending on dose. For example, in patients with schizophrenia, cariprazine exhibited a greater preference for occupying dopamine D_3_ versus D_2_ receptors at lower doses relative to higher doses.^40^ Since D_3_ receptors are preferentially expressed in regions of the brain that are believed to be involved in modulating mood and cognition^21^ ^,^^41^ ^,^^42^, the preferential occupancy of D_3_ receptors at lower doses may contribute to the efficacy of cariprazine on depressive symptoms in bipolar depression, a population where the effective doses of cariprazine are lower than those explored in the bipolar mania studies. At higher doses, D_2_ and D_3_ receptor occupancy is more balanced, ^40^ which may be beneficial in patients with bipolar mania or mixed features. Unlike the depressive symptom scales, there were no dose effects seen in the CGI-S results as cariprazine demonstrated efficacy versus placebo on the CGI-S in all patient and dose groups, indicating an overall and broad treatment benefit by cariprazine in bipolar depression. Taken together, these results suggest that both doses of cariprazine are effective in improving symptoms in patients with bipolar depression, though clinicians should be aware that different subsets of patients may respond differently to higher versus lower doses of cariprazine.

Patients with mixed features have a higher propensity for relapse, recurrence, and nonrecovery, suggesting that an effective treatment in patients with mixed features may also offer a higher probability of sustaining acute benefits. Some treatments for bipolar depression can exacerbate mania symptoms and the presence of mixed features in bipolar depression has been identified as a risk factor, ^43^ further highlighting the need for treatments that do not destabilize mood or induce a manic switch in patients with bipolar depression and mixed features. In this study, rates of treatment-emergent mania and YMRS total score change were comparable between the cariprazine and placebo groups in patients with and without concurrent manic symptoms, indicating that cariprazine does not have an increased risk of inducing manic episodes or worsening manic symptoms while improving depressive symptoms in patients with bipolar depression.

Results from this study need to be interpreted within its limitations. For example, this analysis was not a prospective study but rather a post hoc study of previously conducted clinical trials in patients with bipolar I depression. As such, the inclusion and exclusion criteria of the constituent studies may prevent results from being generalizable to certain patient populations. For example, patients with suicidal behavior and comorbid disorders were excluded. In addition, patients with YMRS total score >10 (MD-53 and MD-54) or >12 (MD-56) were excluded from these studies, which limited the severity of concurrent manic symptoms in this population. As a result, this post hoc study was not meant to be and is not an evaluation of the DSM-5 mixed features criteria, but instead a broader criteria of baseline YMRS total score ≥4 to identify patients with concurrent manic symptoms was used. This definition has been previously used to categorize patients as having mixed features, ^36^ though the clinical relevance of this definition and similar “mixed features” criteria have not been thoroughly investigated or validated prospectively. Additionally, the YMRS total score analyses should also be interpreted cautiously as the YMRS exclusion criteria may limit the ability to assess and detect meaningful changes in YMRS total score.

## Conclusions

In this post hoc study of 3 randomized, double-blind, placebo-controlled trials in patients with bipolar I depression, cariprazine demonstrated efficacy versus placebo in improving depressive symptoms in patient subgroups with and without concurrent manic symptoms. These results suggest that cariprazine may be an appropriate treatment option for patients with bipolar I depression and concurrent manic symptoms, with higher doses potentially more effective in patients with manic symptoms.

## Supplementary material

For supplementary material accompanying this paper visit https://doi.org/10.1017/S1092852919001287. click here to view supplementary material

## References

[1] Grande I , Berk M , Birmaher B , Vieta E . Bipolar disorder. Lancet. 2016; 387(10027): 1561–1572. 26388529 10.1016/S0140-6736(15)00241-X

[2] Miller S , Dell’Osso B , Ketter TA . The prevalence and burden of bipolar depression. J Affect Disord. 2014; 169(Suppl 1): S3–11. 25533912 10.1016/S0165-0327(14)70003-5

[3] Kupka RW , Altshuler LL , Nolen WA , et al. Three times more days depressed than manic or hypomanic in both bipolar I and bipolar II disorder. Bipolar Disord. 2007; 9(5): 531–535. 17680925 10.1111/j.1399-5618.2007.00467.x

[4] Kessler RC , Akiskal HS , Ames M , et al. Prevalence and effects of mood disorders on work performance in a nationally representative sample of U.S. workers. Am J Psychiatry. 2006; 163(9): 1561–1568. 16946181 10.1176/appi.ajp.163.9.1561 PMC1924724

[5] Simon GE , Ludman EJ , Unutzer J , Operskalski BH , Bauer MS . Severity of mood symptoms and work productivity in people treated for bipolar disorder. Bipolar Disord. 2008; 10(6): 718–725. 18837866 10.1111/j.1399-5618.2008.00581.x

[6] American Psychiatric Association. Diagnostic and Statistical Manual of Mental Disorders, 4th ed. Washington, DC: American Psychiatric Association; 1994.

[7] Swann AC , Lafer B , Perugi G , et al. Bipolar mixed states: an international society for bipolar disorders task force report of symptom structure, course of illness, and diagnosis. Am J Psychiatry. 2013; 170(1): 31–42. 23223893 10.1176/appi.ajp.2012.12030301

[8] Rosenblat JD , McIntyre RS . Treatment of mixed features in bipolar disorder. CNS Spectr. 2017; 22(2): 141–146. 27618746 10.1017/S1092852916000547

[9] Fornaro M , Stubbs B , De Berardis D , et al. Atypical antipsychotics in the treatment of acute bipolar depression with mixed features: a systematic review and exploratory meta-analysis of placebo-controlled clinical trials. Int J Mol Sci. 2016; 17(2): 241. 26891297 10.3390/ijms17020241 PMC4783972

[10] American Psychiatric Association. Diagnostic and Statistical Manual of Mental Disorders, 5th ed. Washington, DC: American Psychiatric Association; 2013.

[11] McIntyre RS , Soczynska JK , Cha DS , et al. The prevalence and illness characteristics of DSM-5-defined "mixed feature specifier" in adults with major depressive disorder and bipolar disorder: results from the International Mood Disorders Collaborative Project. J Affect Disord. 2015; 172:259–264. 25451425 10.1016/j.jad.2014.09.026

[12] Miller S , Suppes T , Mintz J , et al. Mixed depression in bipolar disorder: prevalence rate and clinical correlates during naturalistic follow-up in the Stanley bipolar network. Am J Psychiatry. 2016; 173(10): 1015–1023. 27079133 10.1176/appi.ajp.2016.15091119

[13] Perugi G , Angst J , Azorin JM , et al. Mixed features in patients with a major depressive episode: the BRIDGE-II-MIX study. J Clin Psychiatry. 2015; 76(3): e351–e358. 25830457 10.4088/JCP.14m09092

[14] Goldberg JF . Mixed depression: a farewell to differential diagnosis? J Clin Psychiatry. 2015; 76(3): e378–e380. 25830464 10.4088/JCP.14com09578

[15] Koukopoulos A , Sani G . DSM-5 criteria for depression with mixed features: a farewell to mixed depression. Acta Psychiatr Scand. 2014; 129(1): 4–16. 23600771 10.1111/acps.12140

[16] Goldberg JF , Perlis RH , Bowden CL , et al. Manic symptoms during depressive episodes in 1,380 patients with bipolar disorder: findings from the STEP-BD Am J Psychiatry. 2009; 166(2): 173–181. 19122008 10.1176/appi.ajp.2008.08050746 PMC10034853

[17] Betzler F , Stover LA , Sterzer P , Kohler S . Mixed states in bipolar disorder – changes in DSM-5 and current treatment recommendations. Int J Psychiatry Clin Pract. 2017; 21(4): 244–258. 28417647 10.1080/13651501.2017.1311921

[18] Pacchiarotti I , Bond DJ , Baldessarini RJ , et al. The International Society for Bipolar Disorders (ISBD) task force report on antidepressant use in bipolar disorders. Am J Psychiatry. 2013; 170(11): 1249–1262. 24030475 10.1176/appi.ajp.2013.13020185 PMC4091043

[19] Fornaro M , Anastasia A , Novello S , et al. Incidence, prevalence and clinical correlates of antidepressant-emergent mania in bipolar depression: a systematic review and meta-analysis . Bipolar Disord. 2018; 20(3): 195–227. 29441650 10.1111/bdi.12612

[20] Post RM . The impact of bipolar depression. J Clin Psychiatry. 2005; 66 Suppl 5:5–10. 16038596

[21] Leggio GM , Salomone S , Bucolo C , et al. Dopamine D(3) receptor as a new pharmacological target for the treatment of depression. Eur J Pharmacol. 2013; 719(1-3): 25–33. 23872400 10.1016/j.ejphar.2013.07.022

[22] Celada P , Puig M , Amargos-Bosch M , Adell A , Artigas F . The therapeutic role of 5-HT1A and 5-HT2A receptors in depression. J Psychiatry Neurosci. 2004; 29(4): 252–265. 15309042 PMC446220

[23] Stahl SM , Morrissette DA , Faedda G , et al. Guidelines for the recognition and management of mixed depression. CNS Spectr. 2017; 22(2): 203–219. 28421980 10.1017/S1092852917000165

[24] Durgam S , Earley W , Lipschitz A , et al. An 8-week randomized, double-blind, placebo-controlled evaluation of the safety and efficacy of cariprazine in patients with bipolar I depression. Am J Psychiatry. 2016; 173(3): 271–281. 26541814 10.1176/appi.ajp.2015.15020164

[25] Earley W , Burgess M , Rekeda L , et al. Cariprazine treatment of bipolar depression: a randomized double-blind placebo-controlled phase 3 study. Am J Psychiatry. 2019; 176(6): 439–448. 30845817 10.1176/appi.ajp.2018.18070824

[26] Earley W , Burgess M , Khan B , et al. Treatment of bipolar I depression with cariprazine: a randomized double-blind placebo-controlled trial. Poster presented at: Psych Congress; October 25-28, 2018; Orlando, FL.

[27] Hamilton M . A rating scale for depression. J Neurol Neurosurg Psychiatry. 1960; 23(1): 56–62. 14399272 10.1136/jnnp.23.1.56 PMC495331

[28] Hamilton M . Development of a rating scale for primary depressive illness. Br J Soc Clin Psychol. 1967; 6(4): 278–296. 6080235 10.1111/j.2044-8260.1967.tb00530.x

[29] Miller IW , Bishop S , Norman WH , Maddever H . The modified Hamilton rating scale for depression: reliability and validity. Psychiatry Res. 1985; 14(2): 131–142. 3857653 10.1016/0165-1781(85)90057-5

[30] Guy W . Clinical Global Impressions In: Guy W , ed. ECDEU Assessment Manual for Psychopharmacology. Rockville, MD: National Institute of Mental Health, Psychopharmacology Research Branch; 1976:217–222.

[31] Young RC , Biggs JT , Ziegler VE , Meyer DA . A rating scale for mania: reliability, validity and sensitivity. Br J Psychiatry. 1978; 133:429–435. 728692 10.1192/bjp.133.5.429

[32] Posner K , Brown GK , Stanley B , et al. The Columbia-Suicide Severity Rating Scale: initial validity and internal consistency findings from three multisite studies with adolescents and adults. Am J Psychiatry. 2011; 168(12): 1266–1277. 22193671 10.1176/appi.ajp.2011.10111704 PMC3893686

[33] Montgomery SA , Asberg M . A new depression scale designed to be sensitive to change. Br J Psychiatry. 1979; 134:382–389. 444788 10.1192/bjp.134.4.382

[34] Frye MA , Helleman G , McElroy SL , et al. Correlates of treatment-emergent mania associated with antidepressant treatment in bipolar depression. Am J Psychiatry. 2009; 166(2): 164–172. 19015231 10.1176/appi.ajp.2008.08030322

[35] Tohen M , Kanba S , McIntyre RS , Fujikoshi S , Katagiri H . Efficacy of olanzapine monotherapy in the treatment of bipolar depression with mixed features. J Affect Disord. 2014; 164:57–62. 24856554 10.1016/j.jad.2014.04.003

[36] McIntyre RS , Cucchiaro J , Pikalov A , Kroger H , Loebel A . Lurasidone in the treatment of bipolar depression with mixed (subsyndromal hypomanic) features: post hoc analysis of a randomized placebo-controlled trial. J Clin Psychiatry. 2015; 76(4): 398–405. 25844756 10.4088/JCP.14m09410

[37] Patkar A , Gilmer W , Pae CU , et al. A 6 week randomized double-blind placebo-controlled trial of ziprasidone for the acute depressive mixed state. PLoS One. 2012; 7(4): e34757. 22545088 10.1371/journal.pone.0034757 PMC3335844

[38] Judd LL , Schettler PJ , Akiskal H , et al. Prevalence and clinical significance of subsyndromal manic symptoms, including irritability and psychomotor agitation, during bipolar major depressive episodes. J Affect Disord. 2012; 138(3): 440–448. 22314261 10.1016/j.jad.2011.12.046 PMC3677770

[39] McIntyre RS . Mixed features and mixed states in psychiatry: from calculus to geometry. CNS Spectr. 2017; 22(2): 116–117. 28264727 10.1017/S1092852916000559

[40] Girgis RR , Slifstein M , D’Souza D , et al. Preferential binding to dopamine D3 over D2 receptors by cariprazine in patients with schizophrenia using PET with the D3/D2 receptor ligand [(11)C]-(+)-PHNO. Psychopharmacology (Berl). 2016; 233(19-20): 3503–3512. 27525990 10.1007/s00213-016-4382-y PMC5035321

[41] Stahl SM . Stahl’s Essential Psychopharmacology: Neuroscientific Basis and Practical Applications. 4th ed. Cambridge, New York: Cambridge University Press; 2013.

[42] Nakajima S , Gerretsen P , Takeuchi H , et al. The potential role of dopamine D(3) receptor neurotransmission in cognition. Eur Neuropsychopharmacol. 2013; 23(8): 799–813. 23791072 10.1016/j.euroneuro.2013.05.006 PMC3748034

[43] Niitsu T , Fabbri C , Serretti A . Predictors of switch from depression to mania in bipolar disorder. J Psychiatr Res. 2015; 66-67:45–53. 25937504 10.1016/j.jpsychires.2015.04.014

