# Supplementary material for: Cariprazine efficacy in bipolar I depression with and without concurrent manic symptoms: post hoc analysis of 3 randomized, placebo-controlled studies
Source: CNS Spectr. 2019 Oct 2;25(4):502–10. doi: 10.1017/S1092852919001287 (PMC7511904; doi:10.1017/S1092852919001287)
Supplement: Supplementary file 1 [file S1092852919001287sup.zip › S1092852919001287sup002.docx]

Supplemental Figure 2. By-Week Change in CGI-S Score From Baseline to Week 6 in Patients (A) With or (B) Without Manic Symptoms (MMRM)


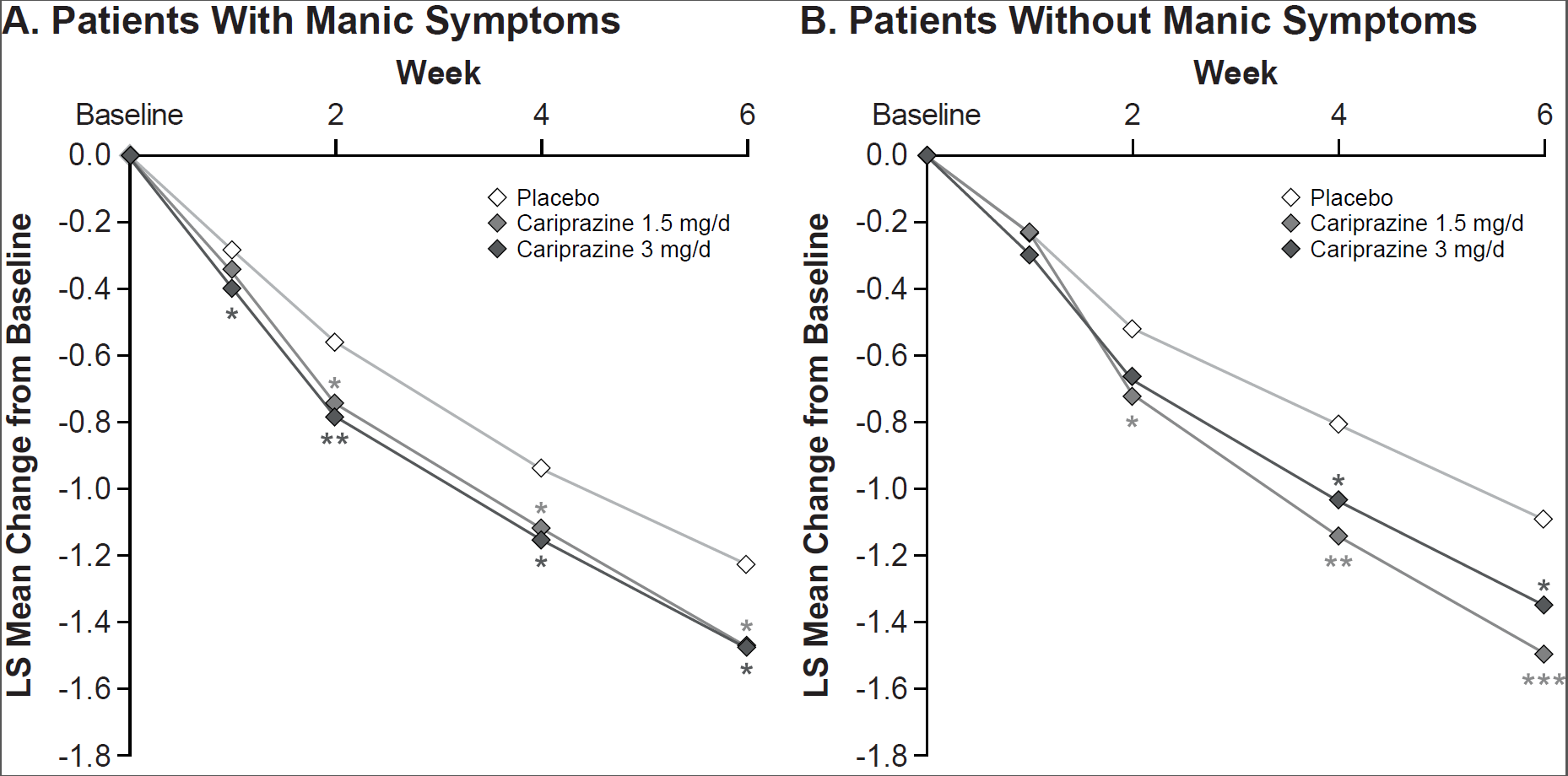


**P*<.05, ***P*<.01, ****P<*.001 vs placebo.

CGI-S, Clinical Global Impressions-Severity Scale; LS, least squares; MMRM, mixed-effects model for repeated measures.
