# Supplementary material for: Cariprazine efficacy in bipolar I depression with and without concurrent manic symptoms: post hoc analysis of 3 randomized, placebo-controlled studies
Source: CNS Spectr. 2019 Oct 2;25(4):502–10. doi: 10.1017/S1092852919001287 (PMC7511904; doi:10.1017/S1092852919001287)
Supplement: Supplementary file 1 [file S1092852919001287sup.zip › S1092852919001287sup003.docx]

Supplemental Table 1. Additional Baseline Characteristics, Safety Population

| Characteristic | **With Manic Symptoms (n=808)** | | | | **Without Manic Symptoms (n=575)** | | |
| --- | --- | --- | --- | --- | --- | --- | --- |
|  |  | Cariprazine | | |  | Cariprazine | |
|  | PBO  (n=267) | | 1.5 mg/d  (n=280) | 3 mg/d  (n=274) | PBO  (n=201) | 1.5 mg/d  (n=190) | 3 mg/d  (n=195) |
| Duration of current depressive episode, mean (SD), mo | 3.8 (2.6) | | 3.8 (2.6) | 3.5 (2.3) | 3.4 (2.5) | 3.7 (2.5) | 3.5 (2.5) |
| Depressive episodes during lifetime, mean (SD), No. | 7.3 (7.6) | | 7.4 (7.3) | 6.8 (8.6) | 6.4 (6.8) | 6.5 (6.5) | 6.7 (8.4) |
| Manic/mixed episodes during lifetime, mean (SD), No. | 4.9 (5.6) | | 4.5 (4.7) | 4.4 (4.4) | 4.2 (5.1) | 3.9 (3.8) | 4.4 (6.0) |
| Mood episodes during past year, mean (SD), No. | 1.6 (0.7) | | 1.7 (0.7) | 1.6 (0.7) | 1.5 (0.6) | 1.5 (0.7) | 1.6 (0.7) |
| PBO, placebo; SD, standard deviation. | | | | | | | |
